# Large-scale engineering of hiPSC-derived nephron sheets and cryopreservation of their progenitors

**DOI:** 10.1186/s13287-022-02881-5

**Published:** 2022-05-16

**Authors:** Loes E. Wiersma, M. Cristina Avramut, Ellen Lievers, Ton J. Rabelink, Cathelijne W. van den Berg

**Affiliations:** 1 Department of Internal Medicine - Nephrology, Leiden University Medical Center, Postal Zone C7-Q, Albinusdreef 2, 2333 ZA Leiden, The Netherlands; 2 Einthoven Laboratory of Vascular and Regenerative Medicine, Leiden University Medical Center, Leiden, The Netherlands; 3 Department of Cell and Chemical Biology - Electron Microscopy, Leiden University Medical Center, Postal zone S-1-P, Einthovenweg 20, 2333 ZC Leiden, The Netherlands

**Keywords:** Induced pluripotent stem cells, Kidney organoids, Kidney transplantation, Scale-up, Engineering, Cryopreservation, Regenerative medicine

## Abstract

**Background:**

The generation of human induced pluripotent stem cells (hiPSCs) has opened a world of opportunities for stem cell-based therapies in regenerative medicine. Currently, several human kidney organoid protocols are available that generate organoids containing kidney structures. However, these kidney organoids are relatively small ranging up to 0.13 cm^2^ and therefore contain a small number of nephrons compared to an adult kidney, thus defying the exploration of future use for therapy.

**Method:**

We have developed a scalable, easily accessible, and reproducible protocol to increase the size of the organoid up to a nephron sheet of 2.5 cm^2^ up to a maximum of 12.6 cm^2^ containing a magnitude of nephrons.

**Results:**

Confocal microscopy showed that the subunits of the nephrons remain evenly distributed throughout the entire sheet and that these tissue sheets can attain ~ 30,000–40,000 glomerular structures. Upon transplantation in immunodeficient mice, such nephron sheets became vascularized and matured. They also show reuptake of injected low-molecular mass dextran molecules in the tubular structures, indicative of glomerular filtration. Furthermore, we developed a protocol for the cryopreservation of intermediate mesoderm cells during the differentiation and demonstrate that these cells can be successfully thawed and recovered to create such tissue sheets.

**Conclusion:**

The scalability of the procedures, and the ability to cryopreserve the cells during differentiation are important steps forward in the translation of these differentiation protocols to future clinical applications such as transplantable auxiliary kidney tissue.

**Supplementary Information:**

The online version contains supplementary material available at 10.1186/s13287-022-02881-5.

## Introduction

More than 10% of the world population suffers from chronic kidney disease [1] that can result in end-stage renal disease (ESRD) in which kidney function is lost. In 2030, an estimated 5.4 million people globally will have ESRD [2], fuelled by an aging population and increase in obesity, diabetes and cardiovascular disease [3]. To cope, ~ 5% of national healthcare budgets would need to be allocated to dialysis and expensive renal replacement therapies [4]. Kidney transplantation is currently the best clinical option but is hampered by organ availability and the use of immune suppressive drugs. These limitations drive the field of regenerative medicine to develop attractive therapies by engineering, replacing or regenerating cells, organs or tissues in order to restore function after damage or loss. The field has seen tremendous growth with the introduction of human induced pluripotent stem cells (hiPSCs) [5] that can differentiate into any cell of the body. Generation of hiPSC-derived human kidney tissues may serve as an future alternative source of kidney tissue that could be used for transplantation purposes to (partially) restore kidney function [6]. In line, we previously demonstrated that transplanting hiPSC-derived kidney organoids under the renal kidney capsule of immunodeficient mice resulted in vascularization, maturation, and functional and size selective glomerular filtration [7, 8].

However, to take this concept into further development towards clinical application, it is essential to demonstrate that the culture of these hiPSC-derived kidney organoids can be scaled to contain meaningful numbers of nephrons. Current dialysis treatment, on average, delivers 10% of the normal glomerular filtration and clearance. We therefore reasoned that a scaled culture system should be able to deliver at least 50,000 glomeruli to be considered as potential future auxiliary transplant tissue. Here we report a method to culture hiPSC-derived nephron tissue in sheets that contain tens of thousands of glomerular structures, while maintaining their ability to be transplanted and become functional. In addition, we show that the intermediate mesoderm cells in the differentiation culture can be cryopreserved thus enabling a structured and planned manufacturing process of these nephron sheets.

## Materials and methods

### hiPSC maintenance

hiPSCs were maintained as previously described [7]. Briefly, all hiPSC lines were cultured in Essential 8 (E8) medium (Life Technologies) on vitronectin (Thermo Fisher Scientific) coated plates (Corning) and passaged twice a week with EDTA (Thermo Fisher Scientific). We used 3 hiPSC lines generated by the hiPSC core facility of the LUMC: LUMC0020iCTRL6.4, LUMC0072iCTRL01 and LUMC0099iCTRL04 that were generated from fibroblasts using Sendai virus [9], RNA (Simplicon RNA Reprogramming Kit, Millipore) and ReproRNA (Stemcell Technologies) respectively (detailed information at Human Pluripotent Stem Cell Registry, https://hpscreg.eu/). Additionally, we used reporter cell line MAFB:mTagBFP2 that was reprogrammed and gene-edited using CRISPR/Cas9 [10]. Cell lines are respectively referred to as LUMC0020, LUMC0072, LUMC0099 and iPSC-MAFB.

### Differentiation of organoids and nephron sheets

Differentiation of organoids was described previously [7, 11]. Briefly, cells were seeded as single cells at variable densities (8000–26,000 cells/cm^2^) per cell line on 6 well plates. Differentiating cells were dissociated on day 7 and centrifuged at 400 × *g* containing 5 × 10^5^ cells per tube. Cell clumps were pipetted on top of a 0.4 µm pore transwell membrane (24 mm, Corning) and further maintained until day 7 + 18.

For differentiation to hiPSC-derived nephron sheets, hiPSCs were dissociated using TrypLE Select (Thermo Fisher Scientific) and plated at similar densities as above on a vitronectin coated T75 (Cellstar) in E8 medium supplemented with Revitacell. Differentiation was started the following day (day 0) by replacing medium with STEMdiff APEL2 Medium (APEL2, Stemcell Technologies) containing 1% PFHMII (Life Technologies), 1% Antibiotic–Antimycotic (Life Technologies) and 8 µM CHIR99021 (Tocris). On day 4 medium was switched to APEL2 supplemented with 200 ng/mL rhFGF9 (R&D Systems) and 1 µg/mL Heparin (Sigma-Aldrich). On day 7 intermediate mesodermal cells were dissociated after a 1 h pulse with 5 µM CHIR in APEL2 using Trypsin–EDTA (Thermo Fisher Scientific). Cells were counted using an automated cell counter (NC-200) and centrifuged at 260 × *g* in 50 mL tubes. Cells were seeded at a density between 19.5–23 × 10^6^ cells/cm^2^ on a 0.4 µm pore transwell (75 mm, Corning) using two different templates for cell distribution. Cells were plated either inside a rubber ring (DWK life science) or overlaid with a silicone cover (GRACE BIO-LABS). Nephron sheet differentiation was continued in APEL2 containing FGF9 and heparin with a media change every other day. On day 7 + 5 the template was removed from the sheet, and medium was changed to plain APEL2. Tissue sheets were maintained until day 7 + 18 before fixation with 2% PFA (Alfa Aesar) diluted in PBS.

### Cryopreservation of differentiating nephron sheets

Differentiating intermediate mesoderm cells from 4 independent differentiation experiments were cryopreserved on day 7. Cells were dissociated after CHIR pulse using Trypsin–EDTA, and single cells were counted and resuspended in ice-cold Nutrifreez (Biological Industries). Cryovials containing 8 × 10^6^ cells/mL were rate controlled (− 1 °C/min) frozen to − 80 °C. 24 h later vials were transferred and stored in liquid nitrogen. For thawing, vials were warmed at 37 °C and cells were transferred to a 50 mL tube containing plain DMEM and counted. Cells were centrifuged at the previously indicated density, resuspended in a small volume of 10% FBS in DMEM and pipetted onto the transwell membrane to allow tissue sheet formation using the templates. The protocol was continued as described above until day 7 + 18.

### Animal experiments

All animal experimental protocols were approved by the animal welfare committee of the Leiden University Medical Center and the Dutch Animal Experiments Committee. To allow for transplantation of nephron sheets, a hollow punch (Renssteig) was used to create a 2 mm circular biopsy. These biopsies were transplanted at day 7 + 17 under the renal capsule of both kidneys in 4 seven-week-old recipient mice (non-obese diabetic/severe combined immunodeficiency (NOD/SCID), Charles River Laboratories). Before sacrifice two mice were anesthetized with isoflurane and injected with low molecular mass 10 kDa dextran labelled with Tetramethylrhodamine (TRITC, TdB Labs). Tissues were collected after 14 days of transplantation and processed for immunohistochemistry or transmission electron microscopy.

### Immunohistochemistry

Organoids, untransplanted and transplanted nephron sheets were processed and stained as described previously [7]. In vitro organoids were fixed in 2% PFA for 20 min at 4 °C before storage in PBS at 4 °C. For further analysis biopsies of nephron sheets were either made with a blade or a hollow punch (3 mm, Renssteig). If required, tissues were embedded in TissueTek (Sakura) and stored at − 80 °C. Transplanted tissues were either immediately snapfrozen in Tissue Tek or fixed in 2% PFA overnight. Fixation of tissues containing fluorescent dextran was followed by 8 h incubation in 30% sucrose (Sigma) in PBS (B-BRAUN) at 4 °C. Transplanted tissues were either stored in PBS or embedded in TissueTek. Tissue sections from embedded samples were made using a cryotome (5–10 µm thick).

For immunofluorescence analysis of in vitro organoids and nephron sheets (whole mount, biopsies or slides), samples were blocked in 10% Donkey Serum (Sigma-Aldrich) and 0.3% TritonX (Sigma Aldrich) in PBS for 2 h at room temperature. Samples were stained with primary antibody in blocking buffer for 24–72 h at 4 °C. For transplanted tissues Mouse on Mouse kit (MOM; Vector Laboratories) was used. Tissues were stained with primary antibodies for nephron structures: NPHS1 (R&D), NPHS2 (Abcam), ECAD (BD), LTL and DBA (Vector Laboratories), CUBN (Thermo Fisher Scientific and Abcam); endothelial cells: CD31 (BD) and MECA-32 (BD); and stromal cells: MEIS1/2/3 (Active Motif) and PDGFRα/β (Abcam). Samples were washed 3 times before adding secondary antibody mix for 2 h at room temperature (Additional file 1: Table S1). Nuclei were occasionally counterstained with Hoechst 33258. Organoids and biopsies from nephron sheets were mounted with ProLongGold (Thermo Fisher Scientific) in 35 mm glass bottom dish with a 14 mm or 22 mm glass diameter (MatTek corporation, Willco Wells), or 50 mm glass bottom dish with a 30 mm glass diameter (MatTek corporation). Our largest tissue sheet was mounted on a glass bottom plate (MatTek corporation). Nephron sheets were topped with 12, 20 or 55 mm coverslip, and left to dry for 24–48 h. Samples were imaged using a White Light Laser Confocal Microscope TCS SP8 (Leica) with LAS-X software 3.5.5 and an Andor Dragonfly Spinning Disk with Fusion 2.2 or higher software. LAS-X Image with 3D module and Imaris 9.5.0 were used to further analyze the data.

### Counting glomerular structures

Organoids and biopsies of nephron sheets were stained for glomerular marker NPHS1 and nuclei with Hoechst. During confocal imaging Z-compensation for Excitation and Detector gain were used to create equal fluorescence intensity throughout the entire sample. NPHS1-positive structures were visualized using Imaris and further analyzed using the application ‘Surfaces’ to determine total volume and individual volume of glomerular structures (Additional file 2: Fig. S1). The number of glomerular structures was calculated in individual images from independent experiments. This number was converted to number of glomerular structures in 3-dimensional organoids and nephron sheets.

### Transmission electron microscopy

For Transmission Electron Microscopy (TEM) analysis, small biopsies of transplanted and non-transplanted nephron sheets were sampled and fixed for 1.5 h at room temperature in 1.5% glutaraldehyde (Electron Microscopy Sciences) in 0.1 M sodium cacodylate buffered solution, with pH 7.4. Samples were subsequently rinsed with sodium cacodylate buffer, fixed in 1% osmium tetroxide (Electron Microscopy Sciences) in 0.1 M sodium cacodylate buffer for 1 h on ice, then washed with sodium cacodylate buffer and dehydrated in a series of 70%, 80%, 90% and 100% ethanol. Next, samples were infiltrated with a mixture of 1:1 Epon LX-112 (Ladd Research) and propylene oxide (Electron Microscopy Sciences) for 1.5 h, followed by infiltration with pure Epon for 2 h. Afterwards, samples were mounted in BEEM capsules (Agar Scientific), embedded in pure Epon and polymerized for 48 h at 60 °C. Ultrathin sections (100 nm) were collected on copper slot grids (Storck Veco BV), covered with formvar film and a 6 nm carbon layer. Sections were contrasted with an aqueous solution of 7% uranyl acetate for 20 min, followed by Reynolds’s lead citrate for 10 min. Imaging was performed at an acceleration voltage of 120 kV on a FEI Tecnai G^2^ Spirit BioTWIN transmission electron microscope (FEI), equipped with an Eagle 4 K slow-scan charge-coupled device camera (FEI). Large virtual slides of glomerular and tubular structures were acquired using automated large-scale data collection and stitching software [12] at 13,000 × and 18,500 × magnification. Aperio ImageScope software (Leica Biosystems) was used for the visualization of the virtual slides.

## Results

### Engineering hiPSC-derived nephron sheets using a template

**Fig. 1:**
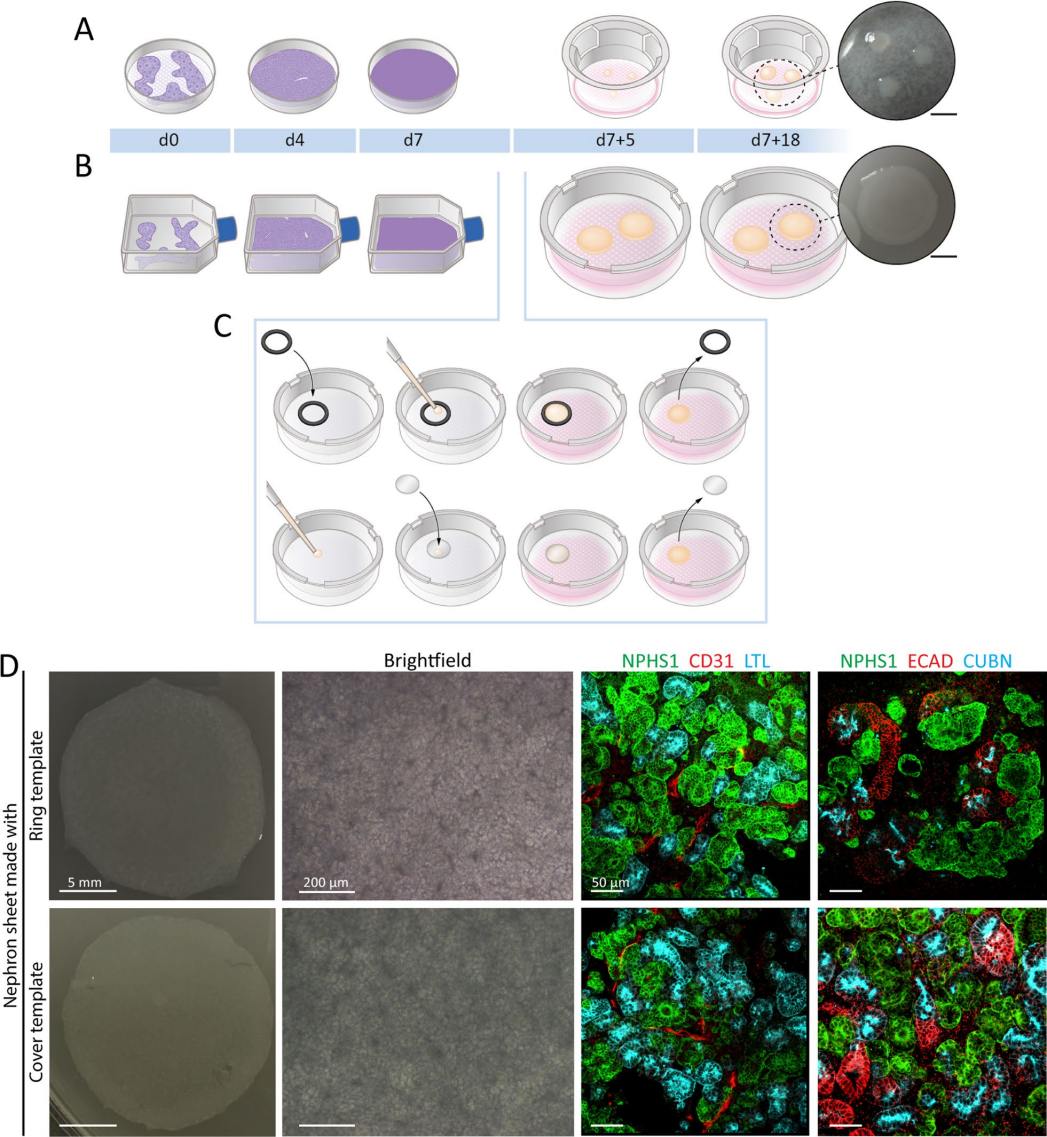
Generating hiPSC-derived nephron sheets using a template. **A** Schematic of the protocol for generating hiPSC-derived kidney organoids. hiPSCs are cultured on multiwell plates as a monolayer until day 7. Cells are dissociated and pipetted as clumps on transwell membranes and cultured until day 7 + 18 (4 mm diameter and 0.13 cm^2^ surface area). Scalebar inset: 5 mm. **B**, **C** Schematic of the protocol for generating hiPSC-derived nephron sheets. hiPSCs are differentiated in T75-culture flasks as a monolayer, followed by dissociation to single cells on day 7. Differentiated cells are either pipetted inside a ring (C, top) or pipetted cells are overlaid with a cover (C, bottom). Nephron sheets are cultured until day 7 + 18 and reach 18 mm diameter with 2.5 cm^2^ surface area. Scalebar inset: 5 mm. **D** Images of whole tissue sheet (left) and brightfield (middle), and immunofluorescence (right) of nephron sheets generated with a ring (top) or cover (bottom)

The original differentiation protocol creates hiPSC-derived kidney organoids with glomerular, proximal tubular and distal tubular structures comparable to human kidney, but these tissues only range up to 4 mm in diameter (Fig. 1A) [7, 11]. Here we focused on designing an easily accessible protocol to generate large hiPSC-derived nephron sheets and aimed to scale-up both the monolayer and 3-dimensional phase. The initial phase of monolayer culture was scaled up to a T75 cell culture flask with comparable seeding density as the original protocol.

**Fig. 2:**
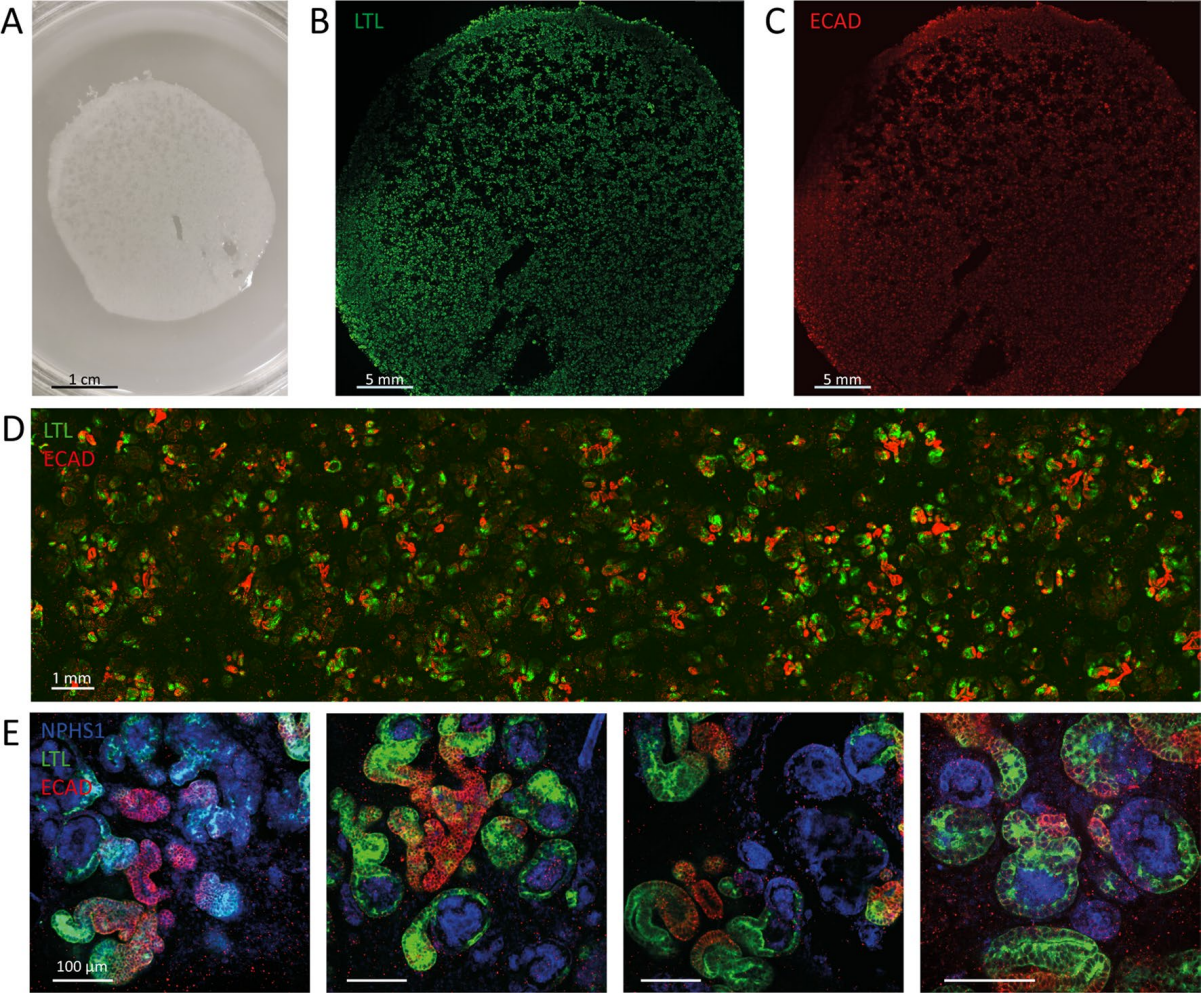
Upscaling of nephron sheets to a diameter of at least 4 cm. **A** Image of large-scale nephron sheet of 12.6 cm^2^ made with a 4 cm diameter cover template (iPSC-MAFB). **B**, **C** Immunofluorescence analysis of the entire nephron sheet shows presence and equal distribution of kidney tubular structures stained for proximal (LTL, B) and distal (ECAD, C) markers. **D** Section of the large nephron sheet highlights the distribution of tubular structures stained for LTL and ECAD combined. **E** Detection of nephron structures after immunofluorescence staining for glomerular structures (NPHS1), proximal tubules (LTL), and distal tubules (ECAD)

Expansion of the 3-dimensional phase of differentiation was performed on a transwell membrane of 7.5 cm diameter. On these transwell membranes templates are necessary to enforce a restricted culture site for standardized seeding and reproducibility. We made use of 2 templates: a rubber ring with an inner circle of 18 mm and a silicone cover of 18 mm (Fig. 1B, C). Both templates yielded similar nephron sheets that are indistinguishable by eye, structures observed in brightfield imaging, and expression of kidney markers by immunofluorescence analysis (Fig. 1D). We therefore combined results from both templates in this study. With these templates we created nephron sheets with a surface area of ~ 2.5 cm^2^, while organoids only have a surface area of ~ 0.13 cm^2^. After 5 days tissue sheets were firm enough to allow for the template to be removed. Sheets further developed in the same manner as the regular organoids. We explored the ability to generate large nephron sheets even further by using a 4 cm silicone cover (Fig. 2A–E). This allowed us to create a tissue sheet with a surface area of 12.6 cm^2^ that showed similar characteristics as the 2.5 cm^2^ as observed by eye and microscopy (Fig. 2A).

### Reproducible hiPSC-derived nephron sheets contain kidney structures

**Fig. 3:**
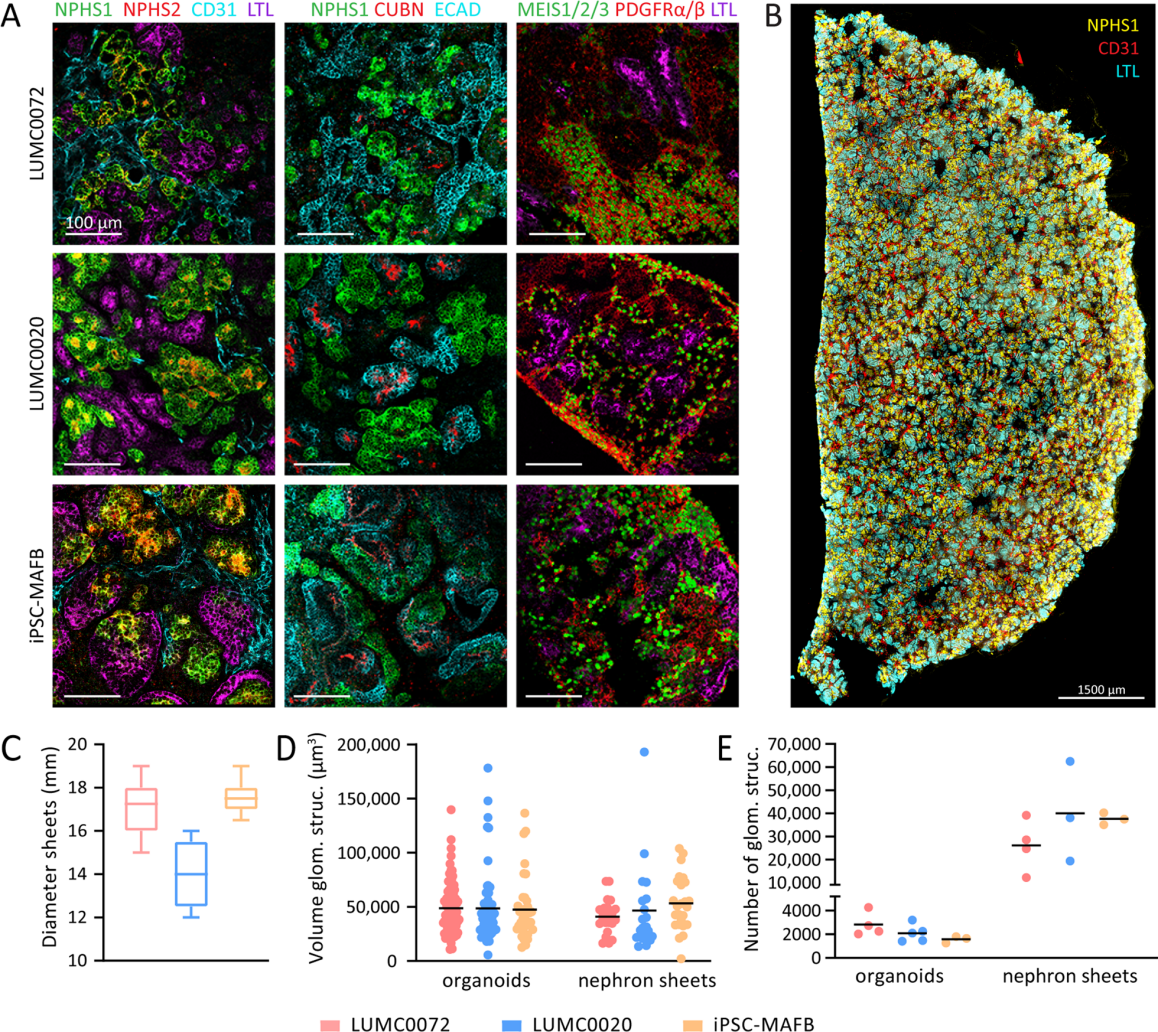
hiPSC-derived nephron sheets reproducibly contain kidney structures and show high number of glomerular structures. **A** Immunofluorescence analysis for glomerular structures (NPHS1, NPHS2), endothelium (CD31), proximal tubule (LTL, CUBN), distal tubular and collecting duct structures (ECAD) in whole mount nephron sheets, and stromal cells (MEIS1/2/3, PDGFRα/β) in cryosections using LUMC0072, LUMC0020 and iPSC-MAFB (representative images from 3 independent experiments). **B** Immunofluorescent 3-dimensional overview showing distribution of NPHS1, CD31 and LTL in bisected hiPSC-derived nephron sheet. **C** Diameter of hiPSC-derived nephron sheets using an 18 mm template in multiple hiPSC lines. (LUMC0072: 32 nephron sheets in 11 independent experiments, LUMC0020: 5 nephron sheets in 3 independent experiments, iPSC-MAFB: 11 nephron sheets in 6 independent experiments). **D** Volume of individual glomerular structures (µm^3^) of organoids and nephron sheets. Each dot represents the volume of a single glomerular structure determined in LUMC0072: 7 organoids from 4 independent experiments and 3 nephron sheets from 3 independent experiments; LUMC0020: 6 organoids in 6 independent experiments and 3 nephron sheets in 3 independent experiments; iPSC-MAFB: 4 organoids in 4 independent experiments and 3 nephron sheets in 3 independent experiments. Bar displays average. **E** Number of glomerular structures in organoids and nephron sheets. Total glomerular number was determined in LUMC0072: 4 organoids from 4 independent experiments and 4 nephron sheets from 4 independent experiments; LUMC0020: 5 organoids in 5 independent experiments and 3 nephron sheets in 3 independent experiments; iPSC-MAFB: 3 organoids in 3 independent experiments and 3 nephron sheets in 3 independent experiments. Bar displays average

To investigate the presence of kidney structures in hiPSC-derived nephron sheets, samples were analyzed for glomerular structures (NHPS1 and NPHS2), endothelial cells (CD31), proximal tubules (LTL, CUBN), distal tubular structures (ECAD), and stromal cells (MEIS1/2/3 and PDGFRα/β) using confocal microscopy. Nephron sheet formation was reproducible in 4 different hiPSC lines (Fig. 3A and Additional file 3: Fig. S2A–D) and renal structures were found throughout the entire sheet (Fig. 3B, Additional file 4: Fig. S3A–C). Our largest nephron sheet (12.6 cm^2^) also showed kidney markers of interest throughout the sheet (Fig. 2B–E). Immunofluorescence analysis was compared to organoids and adult human kidney tissue slides (Additional file 5: Fig. S4).

### Assessment of hiPSC-derived nephron sheets and counting glomerular structures

Nephron sheet diameter was comparable in LUMC0072, LUMC0099 and iPSC-MAFB, while LUMC0020 yielded slightly smaller sheets (Fig. 3C and Additional file 3: Fig. S2B). We aimed to assess the number of glomerular structures (NPHS1^+^) in these tissue sheets and organoids from multiple independent experiments by using the size of individual glomerular structures and their distribution. The size of individual glomerular structures was determined by immunofluorescence analysis with Imaris. The sizes were comparable between cell lines, and organoid and nephron sheet (Fig. 3D and Additional file 3: Fig. S2C). Total number of glomerular structures was evaluated and the average number of structures in organoids ranged between ~ 1300 and 4000 structures, and between ~ 30,000 and 40,000 in nephron sheets (Fig. 3E and Additional file 3: Fig. S2D).

### hiPSC-derived nephron sheets become vascularized and mature upon transplantation

**Fig. 4:**
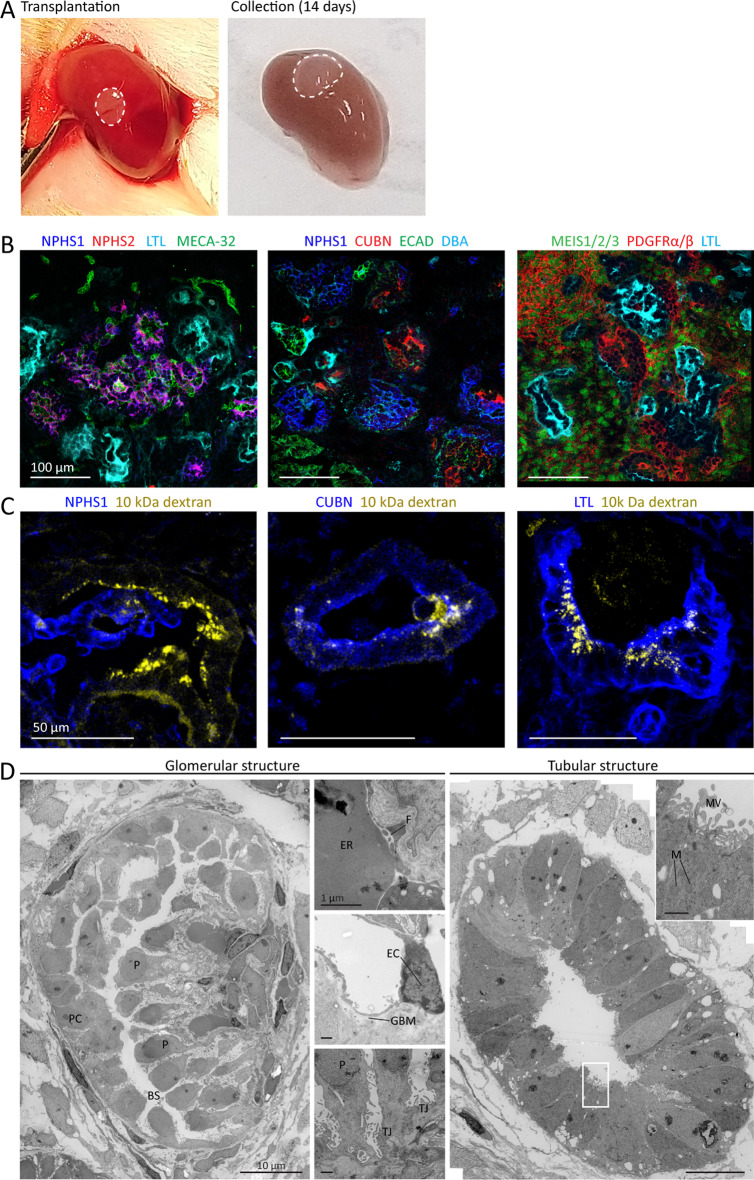
hiPSC-derived nephron sheet become vascularized and mature upon transplantation. **A** Macroscopic images of transplanted biopsy of hiPSC-derived nephron sheet (dotted lines, LUMC0072, day 7 + 17) under renal capsule of mice on the day of transplantation (left) and upon collection after 14 days (right). **B** Immunofluorescence analysis of cryosections demonstrates presence of glomerular structures (NPHS1, NPHS2), mouse endothelial cells (MECA-32), proximal tubules (LTL, CUBN), distal tubules and collecting duct (ECAD, DBA), and stromal cells (MEIS 1/2/3, PDGFRα/β). **C** Detection of intravenously injected low molecular mass dextran (10 kDa, TRITC labeled) combined with immunofluorescence analysis of glomerular (NPHS1) and proximal tubular (CUBN and LTL) on cryosections demonstrates functional filtration. **D** Transmission electron micrographs of a glomerular structure and proximal tubule after transplantation. The glomerular structure shows development of a Bowman’s capsule and podocyte orientation towards a capillary (left image), erythrocytes and fenestrae are lining the blood vessel wall (top small image), smoothing of the glomerular basement membrane and endothelial cells are observed (middle small image) and tight junctions connect podocytes (bottom small image). Proximal tubule shows a single layer of epithelial cells, basal orientation of nuclei, mitochondria, and displays brush border with microvilli. P, podocytes; BS, Bowman’s space; PC, parietal cells; ER, erythrocytes; F, fenestrae; TJ, tight junctions; EC, endothelial cell; GBM, glomerular basement membrane; MV, microvilli; N, nuclei; M, mitochondria

To evaluate whether nephron sheets vascularized and further matured upon transplantation, they were cultured until d7 + 17 and transplanted in mice (Fig. 4A). Due to space limitations under the renal capsule, we transplanted a biopsy of 2 mm of the tissue sheet. After two weeks, before sacrifice, 2 mice were intravenously injected with 10 kDa TRITC-labeled dextran. Confocal imaging showed the presence of all kidney structures and vascularization of the nephron sheet (Fig. 4B and Additional file 6: Fig. S5). Furthermore, TRITC-labeled dextran was observed in glomerular structures demonstrating vascular connection with the host and in the tubular structures indicating functional filtration in glomerular structures (Fig. 4C). Additionally, TEM-images of the transplanted iPSC-derived nephron sheets revealed endothelial cells and erythrocytes in the glomerular structures and maturation was further shown by the presence of fenestrae, open blood vessels, development of the glomerular basement membrane, and foot processes. Tubular structures had an open lumen, a single layer of epithelial cells, mitochondria, a brush border with microvilli, and displayed basal migration of the nuclei (Fig. 4D). Glomerular structures in untransplanted nephron sheets showed formation of Bowman’s space and centered glomerular basement membrane anticipating vascularization. The tubular structures had open and closed lumens but were more disorganized than their transplanted counterparts (Additional file 7: Fig. S6).

### hiPSC-derived nephron sheets can be cryopreserved during differentiation

**Fig. 5:**
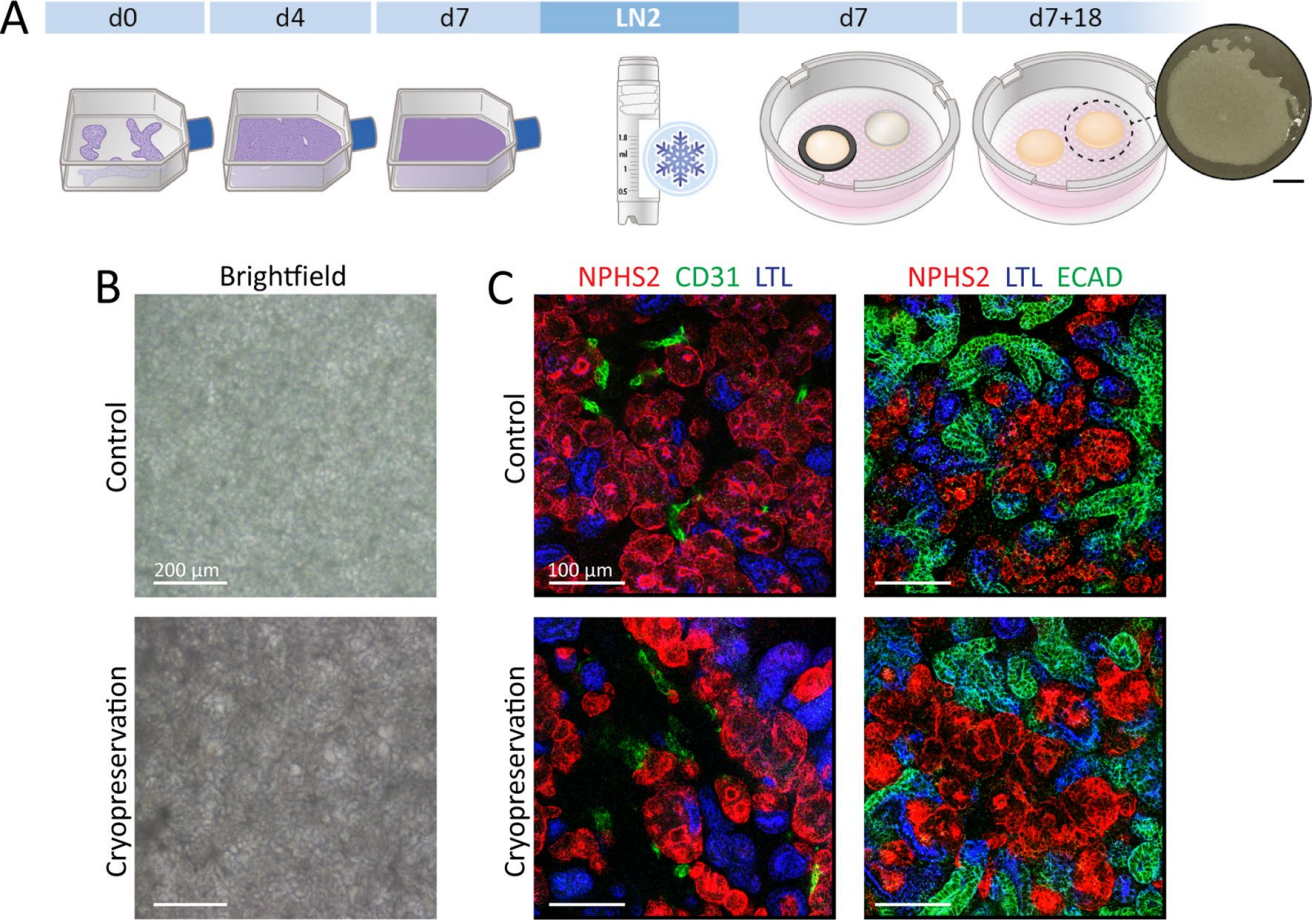
Cryopreservation of hiPSC-derived nephron sheets. **A** Schematic of cryopreservation procedure. hiPSCs are differentiated until day 7 of differentiation, dissociated to single cells and cryopreserved. Cells are thawed and differentiation can be continued using both templates for hiPSC-derived nephron sheets. **B** Brightfield images of nephron sheets without (control) and with cryopreservation (representative image of 4 independent experiments). **C** Immunofluorescence analysis of glomerular structures (NPHS1, NPHS2), endothelium (CD31), proximal tubules (LTL, CUBN), distal tubular structures (ECAD) in whole mount control and cryopreserved nephron sheets (4 independent experiments)

To explore if nephron sheets could be cryopreserved during differentiation, we froze cells at day 7 of differentiation (intermediate mesoderm phase) in cGMP-manufactured cryopreservation medium (Fig. 5A). Nutrifreez provided excellent recovery and tissue sheets were able to continue differentiation after cryopreservation (Fig. 5B, Additional file 8: Fig. S7). Non-frozen and thawed sheets were compared under identical experimental conditions and immunofluorescence showed the presence of glomerular, proximal and distal tubular structures and endothelial cells (Fig. 5C).

## Discussion

Here we engineered a reproducible, scalable hiPSC-derived nephron sheet that can be easily implemented, and show that cryopreservation during differentiation allows for storage of intermediate mesoderm cells, containing progenitors of the kidney. These tissue sheets showed presence of kidney structures throughout with high number of glomerular like structures, and vascularization and maturation upon transplantation under the renal capsule in mice. As observed in previous transplantation studies [8, 13], filtration in glomerular structures was demonstrated by presence of low molecular mass dextran molecules in tubular structures.

Currently multiple protocols are available for designing iPSC-derived cell or tissue sheets, such as retinal pigment epithelium cells on collagen I, smooth muscle like cells on degradable hydrogel, and various cell types on temperature responsive cell culture surfaces like cardiac tissue and hepatocyte-like cells [14–17]. Some methods yield a single sheet layer, while others are stacked to create a multi-layered sheets [18, 19]. Lawlor et al. explored the use of a bioprinter to generate hiPSC-derived kidney organoids [20]. Their printed organoid patch (6 × 8 mm) consisted of a 3-dimensional layer. Our protocol does not require a bioprinter and it is designed to combine manual pipetting and a template to guide nephron sheet growth and patterning. The template creates an optimal ‘cell to square surface’ ratio of differentiating cells resulting in a 3-dimensional sheet. Without a pre-defined culture area, cell density is suboptimal, resulting in an uneven and poor differentiation. Nephron sheets made by either template showed minimal differences in distribution of structures. The templates are easy to use and can be removed without damaging the sheet, allowing further development of the tissue sheet. These nephron sheets can easily be moved from the transwell filter and are strong enough to be transferred without breaking. Additionally, it is important to note that shape and size can be adapted, at least up to 12.6 cm^2^. For clinical purposes, designing an industrialized process for tissue sheet culture could include automated hiPSC-maintenance and differentiation during the monolayer phase, for example in a bioreactor or a multi-layered flask. After harvesting, cells could be further differentiated or cryopreserved. A combination of multiple systems combining hiPSC culture and nephron sheet differentiation could result in an engineered nephron sheet platform.

At present the only treatment for reduced kidney function is dialysis and kidney transplantation. These tissue sheets could potentially fill a gap that alleviates or delays the need for such therapy. We counted glomerular structures in the tissue sheets as an indication for the amount of nephrons that can be transplanted. We found that the average sheets made with an 18 mm template gave rise to tens of thousands of structures, compared to one tenth of these structures in organoids. The latter deviates from previous findings [11] where 500 structures were counted in organoids. Takasato et al. counted structures manually in a 2D image while our method relies on 3D-imaging techniques using the total volume of structures providing a more accurate indication of the number of glomerular structures. With these numbers of glomerular structures in nephron sheets, we hypothesize that transplanting multiple sheets could potentially partially restore kidney function in patients.

To further apply these cultures for research and clinical applications, cryopreservation of differentiating cells is of great interest. We show efficient cryopreservation of differentiating kidney progenitors which alleviates the need for continuous hiPSCs culture and differentiation, and gives rise to an accessible stock of cells. Cryopreservation will also allow for quality control screening on each batch of differentiating organoids. We show that nephron sheets displayed no difference in brightfield imaging and presence of kidney structures upon cryopreservation compared to continuously cultured sheets. Similarly, Mae et al. cryopreserved progenitors of induced ureteric bud organoids and found that they had the same potential to form nephric ducts epithelial aggregates as those who were not cryopreserved [21]. Other groups have shown that cryopreservation of hiPSC-derived cardiomyocytes and hiPSC-blood brain barrier microvascular endothelial cells had little to no adverse effect compared to differentiated cells that did not undergo cryopreservation [22, 23]. These and our findings show great promise for the use of cryopreservation and applicability of these differentiated cells. An additional advantage of the cryopreservation medium used in this study is that it is manufactured under cGMP conditions and allows for clinical translation to a GMP-compliant manufacturing protocol.

## Conclusion

We have focused on developing an easily accessible, robust and efficient culture and cryopreservation method for creating large scale nephron sheets of high quality. These results constitute an important step to future application of these sheets as auxiliary tissue in the treatment of kidney disease in patients. While substantial challenges remain for safety, quality control and correct patterning of these structures, our findings are an advancement in the field of regenerative medicine.

## Supplementary Information

**Additional file 1: Table S1.** Primary and secondary antibodies. Detailed information about primary and secondary antibodies used for immunofluorescence analysis.

**Additional file 2: Fig. S1.** Procedure to calculate total volume of glomerular structures in organoids and nephron sheets. Z-stack imaged by confocal microscopy and imported in Imaris. Absolute intensity is set to detect glomerular (NPHS1+) structures and voxel threshold is used to reduce background noise to provide a clean image. Volume-data on the detected structures is transported to excel for further analysis.

**Additional file 3: Fig. S2.** hiPSC-derived nephron sheets derived from LUMC0099. A Immunofluorescence analysis for glomerular structures (NPHS1, NPHS2), endothelium (CD31), proximal tubule (LTL, CUBN), distal tubular and collecting duct structures (ECAD) in whole mount nephron sheets, and stromal cells (MEIS1/2/3, PDGFRα/β) in cryosections (representative images from 2 independent experiments). B Diameter of hiPSC-derived nephron sheets using an 18 mm template in LUMC0099: 6 nephron sheets in 2 independent experiments. C Volume of individual glomerular structures (µm^3^) of organoids and nephron sheets. Each dot represents the volume of a single glomerular structure determined in LUMC0099: 5 organoids in 2 independent experiments and 2 nephron sheets in 2 independent experiments. Bar displays average. D Number of glomerular structures of organoids and nephron sheets. Glomerular number in organoids was determined: 2 organoids from 2 independent experiments and 3 nephron sheets from 2 independent experiments.

**Additional file 4: Fig. S3.** Immunofluorescent overview of whole or bisected hiPSC-derived nephron sheets. Overview of glomerular (NPHS1), proximal tubular (LTL), distal tubular (ECAD) structures, and basement membrane (LAMININ) in whole nephron sheet (LUMC0072). A Overview of glomerular structures (NPHS1), endothelial cells (CD31) and nuclei (HOECHST) in bisected nephron sheet (LUMC0072). B Overview of distal tubule (ECAD), proximal tubule (LTL) and basement membrane (LAMININ) in bisected nephron sheet (LUMC0020).

**Additional file 5: Fig. S4.** Immunofluorescence analysis of human kidney tissue slides and kidney organoids. Immunofluorescence analysis for glomerular structures (NPHS1, NPHS2), endothelium (human CD31), proximal tubule (LTL, CUBN), distal tubular structures (ECAD), and stromal cells (MEIS1/2/3, PDGFRα/β) in whole kidney organoids, and cryosections of kidney organoids and human kidney.

**Additional file 6: Fig. S5.** Overview of transplanted hiPSC derived nephron sheet. Transplanted biopsy from hiPSC-derived nephron sheet (dotted line, LUMC0072) on the kidney of the recipient mouse. The antibodies for glomerular (NPHS2) and tubular structures (LTL) also recognize these structures in the mouse kidney, but the morphology is different and the hiPSC-derived tissues can be well distinguished from the mouse kidney. MECA-32 only stains mouse endothelial cells.

**Additional file 7: Fig. S6.** hiPSC-derived nephron sheets in vitro at d7 + 31 lack vascularization and are less mature. Transmission electron micrographs of a glomerular and tubular structure in untransplanted nephron sheet. Glomerular structure shows formation of Bowman’s space and centered podocytes anticipating vascularization. The tubular structure is open and epithelial cells are disorganized.

**Additional file 8: Fig. S7.** Overview of nephron sheets without and with cryopreservation. Brightfield images of hiPSC-derived nephron sheets without (control) and after cryopreservation.

## Data Availability

No datasets were generated or analyzed during the current study.

## Abbreviations

CUBN: Cubilin
DBA: Dolichos biflorus agglutinin
E8: Essential 8
ECAD: E-cadherin
ESRD: End stage renal disease
hiPSC: Human induced pluripotent stem cells
kDa: Kilodalton
LTL: Lotus tetragonolobus lectin
MECA: Mouse panendothelial cell antigen
MEIS: Myeloid ecotropic viral integration site
MOM: Mouse on mouse
NPHS1: Nephrin 1
NPHS2: Podocin
NOD/SCID: Non-obese diabetic/severe combined immunodeficiency
PDGFR: Platelet-derived growth factor receptor
TEM: Transmission electron microscopy
TRITC: Tetramethylrhodamine
