# Supplementary material for: Large-scale engineering of hiPSC-derived nephron sheets and cryopreservation of their progenitors
Source: Stem Cell Res Ther. 2022 May 16;13:208. doi: 10.1186/s13287-022-02881-5 (PMC9109372; doi:10.1186/s13287-022-02881-5)
Supplement: Supplementary file 1 — Additional file 1: Table S1. Primary and secondary antibodies. Detailed information about primary and secondary antibodies used for immunofluorescence analysis. [file 13287_2022_2881_MOESM1_ESM.docx]

**Supplemental Table 1. Primary and secondary antibodies**

| Primary antibody | Company | Reference number |
| --- | --- | --- |
| CD31 | BD Pharmingen | 555444 |
| E-cadherin (ECAD) | BD Pharmingen | 610181 |
| Cubilin (CUBN) | Thermo Fisher Scientific | PA5-83684 |
| Cubilin (CUBN) | Abcam | ab191073 |
| DBA | Vector Laboratories | B-1035 |
| LTL | Vector Laboratories | B-1325 |
| MECA-32 | BD Pharmingen | 553849 |
| MEIS1/2/3 | Active Motif | 39795 |
| NPHS1 | R&D Systems | AF4269 |
| NPHS2 | Abcam | ab50339 |
| PDGFRβ | Abcam | ab32570 |

| Secondary antibody | Company | Reference number |
| --- | --- | --- |
| Streptavidin Alexa 405 | Thermo Fisher Scientific | S32351 |
| Streptavidin Alexa 532 | Thermo Fisher Scientific | S11224 |
| Donkey anti Mouse 488 | Thermo Fisher Scientific | A21202 |
| Donkey anti Mouse 568 | Abcam | Ab175700 |
| Donkey anti Mouse 647 | Life Technologies | A31571 |
| Donkey anti Sheep 405 | Abcam | AB175676 |
| Donkey anti Sheep 488 | Thermo Fisher Scientific | A11015 |
| Donkey anti Sheep 568 | Thermo Fisher Scientific | A21099 |
| Donkey anti Rabbit 405 | Abcam | Ab175649 |
| Donkey anti Rabbit 647 | Thermo Fisher Scientific | A31573 |
| Donkey anti Rat 488 | Thermo Fisher Scientific | A21208 |
