# Supplementary figures and images for: Large-scale engineering of hiPSC-derived nephron sheets and cryopreservation of their progenitors
Source: Stem Cell Res Ther. 2022 May 16;13:208. doi: 10.1186/s13287-022-02881-5 (PMC9109372; doi:10.1186/s13287-022-02881-5)

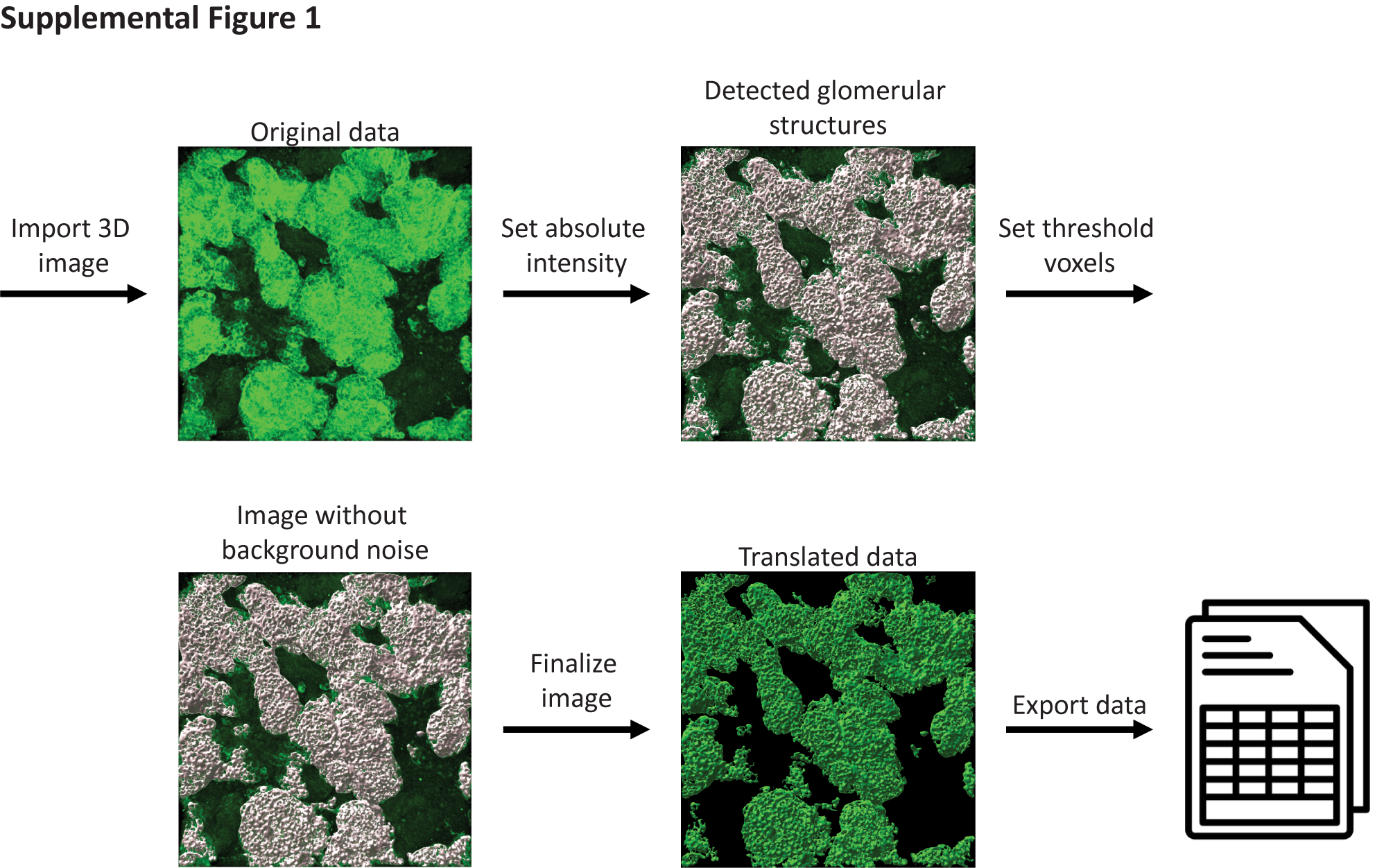

Supplement: Supplementary file 2 — Additional file 2: Fig. S1. Procedure to calculate total volume of glomerular structures in organoids and nephron sheets. Z-stack imaged by confocal microscopy and imported in Imaris. Absolute intensity is set to detect glomerular (NPHS1+) structures and voxel threshold is used to reduce background noise to provide a clean image. Volume-data on the detected structures is transported to excel for further analysis. [file 13287_2022_2881_MOESM2_ESM.tif]

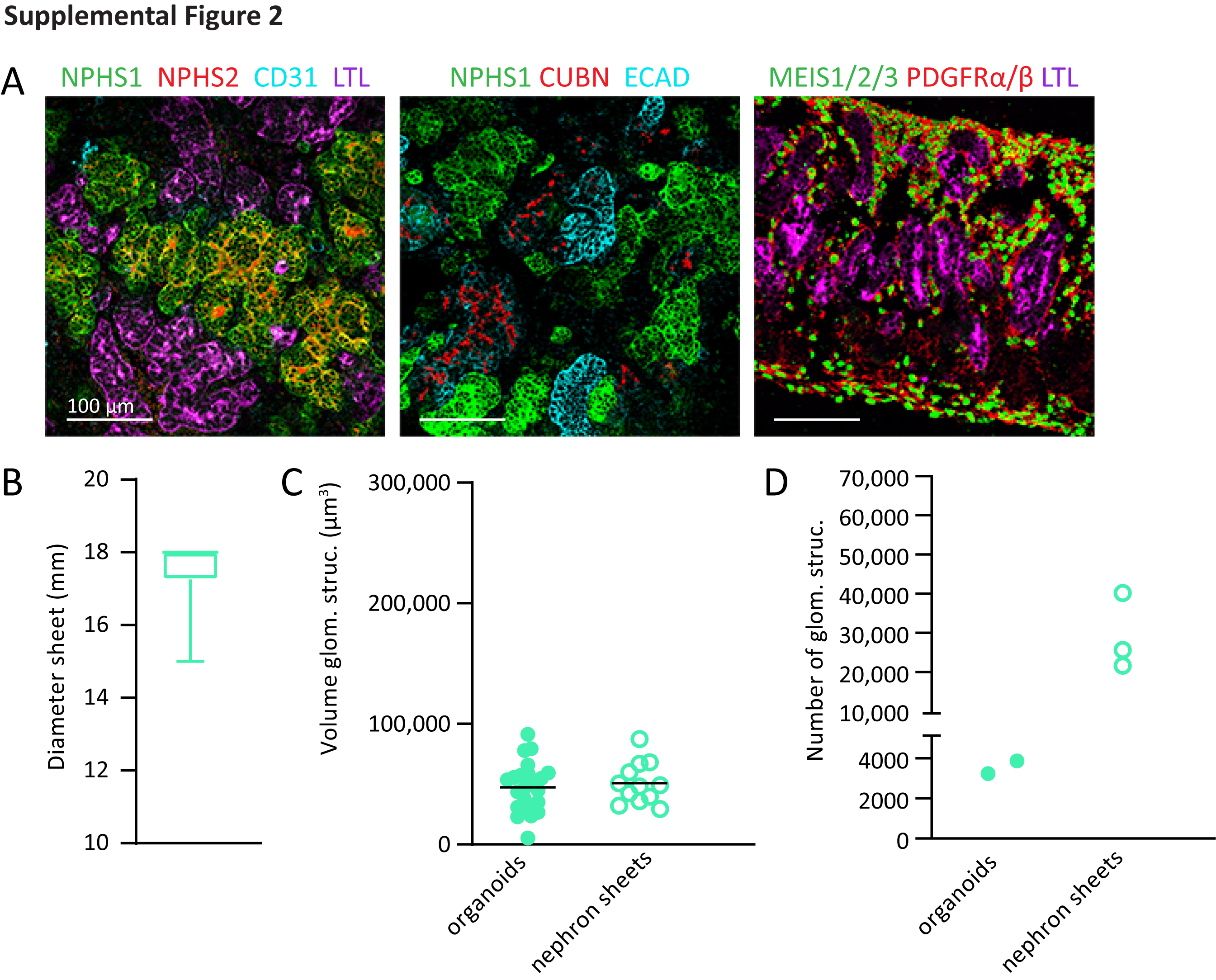

Supplement: Supplementary file 3 — Additional file 3: Fig. S2. hiPSC-derived nephron sheets derived from LUMC0099. A Immunofluorescence analysis for glomerular structures (NPHS1, NPHS2), endothelium (CD31), proximal tubule (LTL, CUBN), distal tubular and collecting duct structures (ECAD) in whole mount nephron sheets, and stromal cells (MEIS1/2/3, PDGFRα/β) in cryosections (representative images from 2 independent experiments). B Diameter of hiPSC-derived nephron sheets using an 18 mm template in LUMC0099: 6 nephron sheets in 2 independent experiments. C Volume of individual glomerular structures (µm3) of organoids and nephron sheets. Each dot represents the volume of a single glomerular structure determined in LUMC0099: 5 organoids in 2 independent experiments and 2 nephron sheets in 2 independent experiments. Bar displays average. D Number of glomerular structures of organoids and nephron sheets. Glomerular number in organoids was determined: 2 organoids from 2 independent experiments and 3 nephron sheets from 2 independent experiments. [file 13287_2022_2881_MOESM3_ESM.tif]

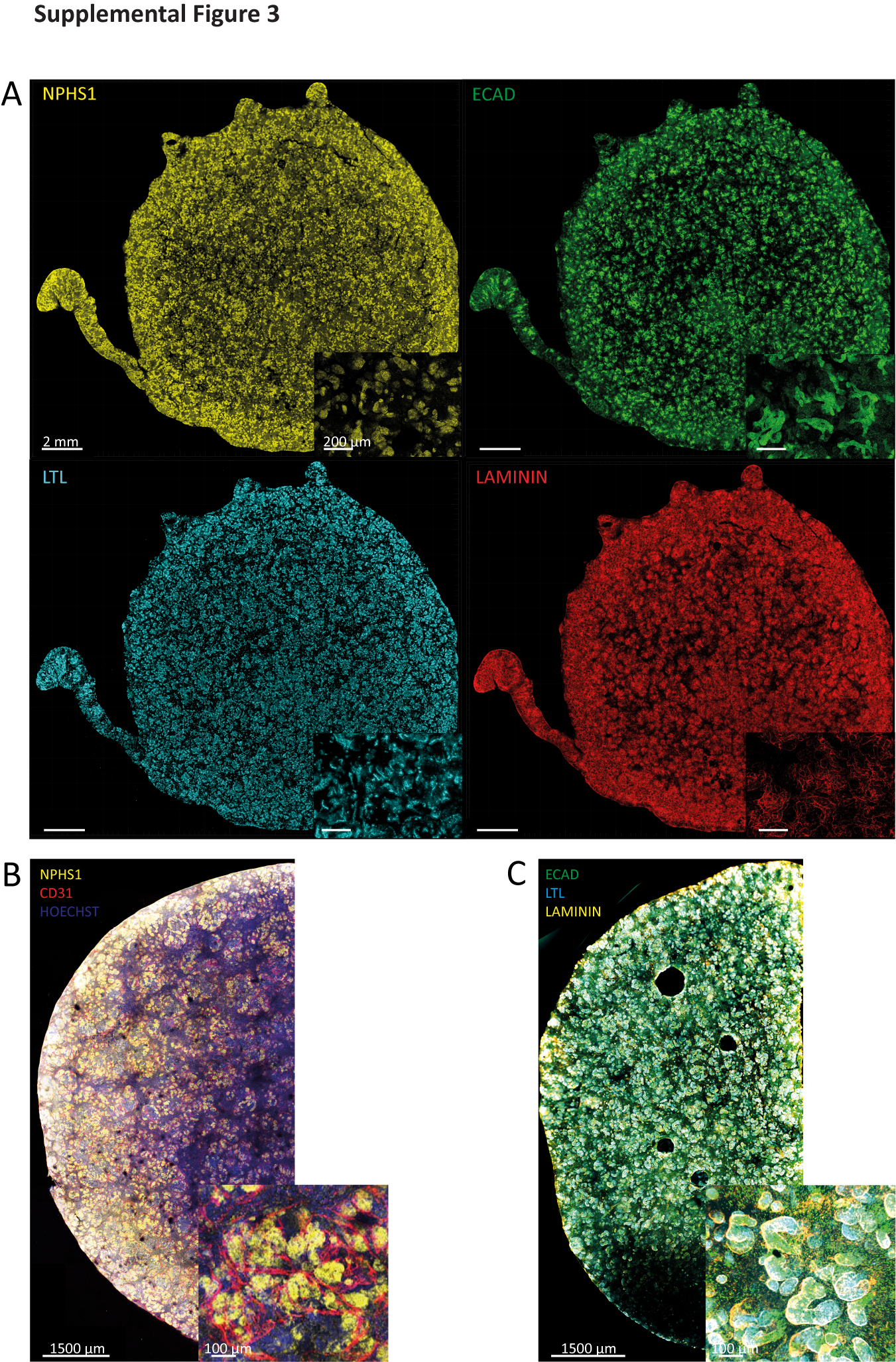

Supplement: Supplementary file 4 — Additional file 4: Fig. S3. Immunofluorescent overview of whole or bisected hiPSC-derived nephron sheets. Overview of glomerular (NPHS1), proximal tubular (LTL), distal tubular (ECAD) structures, and basement membrane (LAMININ) in whole nephron sheet (LUMC0072). A Overview of glomerular structures (NPHS1), endothelial cells (CD31) and nuclei (HOECHST) in bisected nephron sheet (LUMC0072). B Overview of distal tubule (ECAD), proximal tubule (LTL) and basement membrane (LAMININ) in bisected nephron sheet (LUMC0020). [file 13287_2022_2881_MOESM4_ESM.tif]

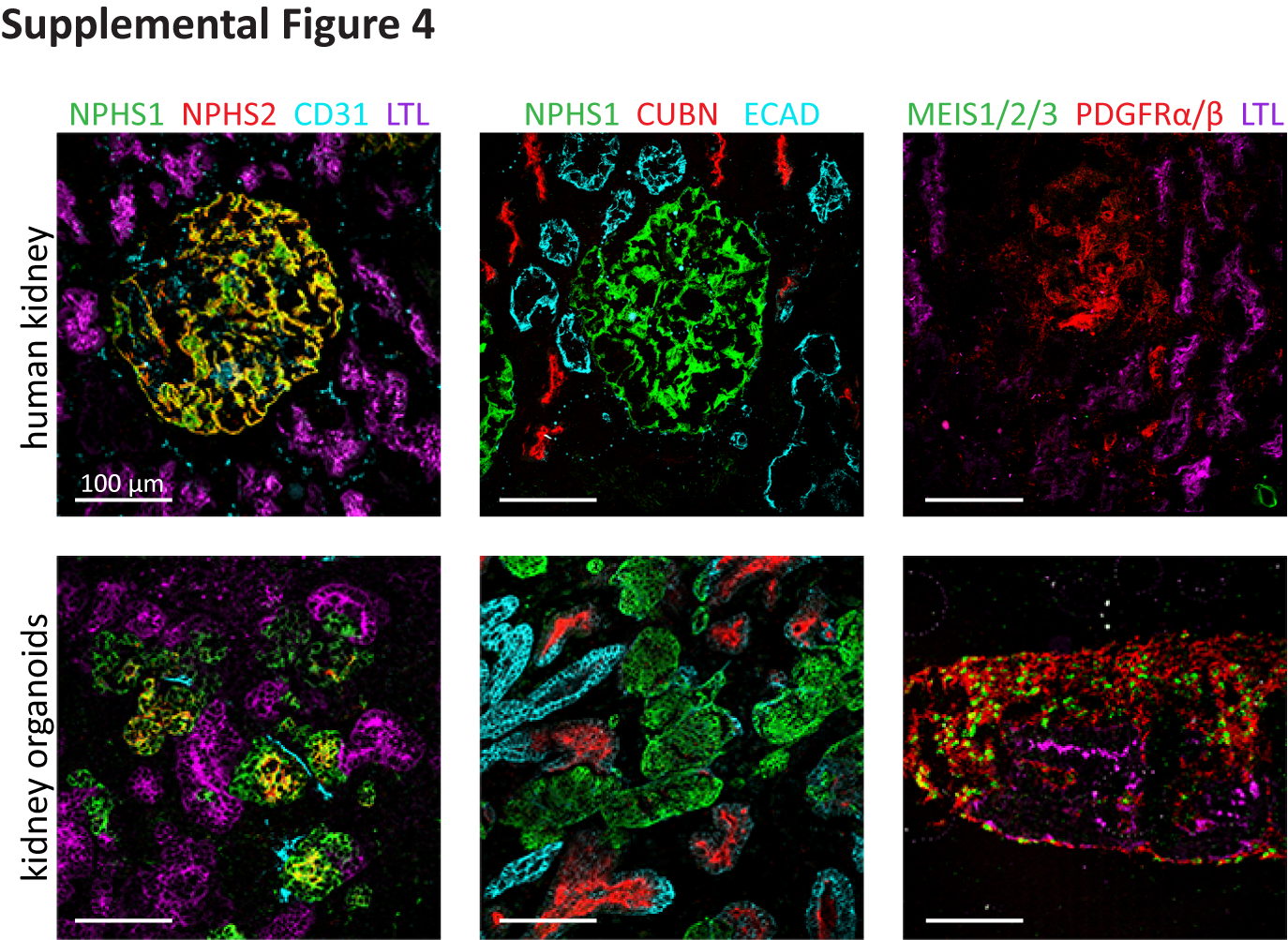

Supplement: Supplementary file 5 — Additional file 5: Fig. S4. Immunofluorescence analysis of human kidney tissue slides and kidney organoids. Immunofluorescence analysis for glomerular structures (NPHS1, NPHS2), endothelium (human CD31), proximal tubule (LTL, CUBN), distal tubular structures (ECAD), and stromal cells (MEIS1/2/3, PDGFRα/β) in whole kidney organoids, and cryosections of kidney organoids and human kidney. [file 13287_2022_2881_MOESM5_ESM.tif]

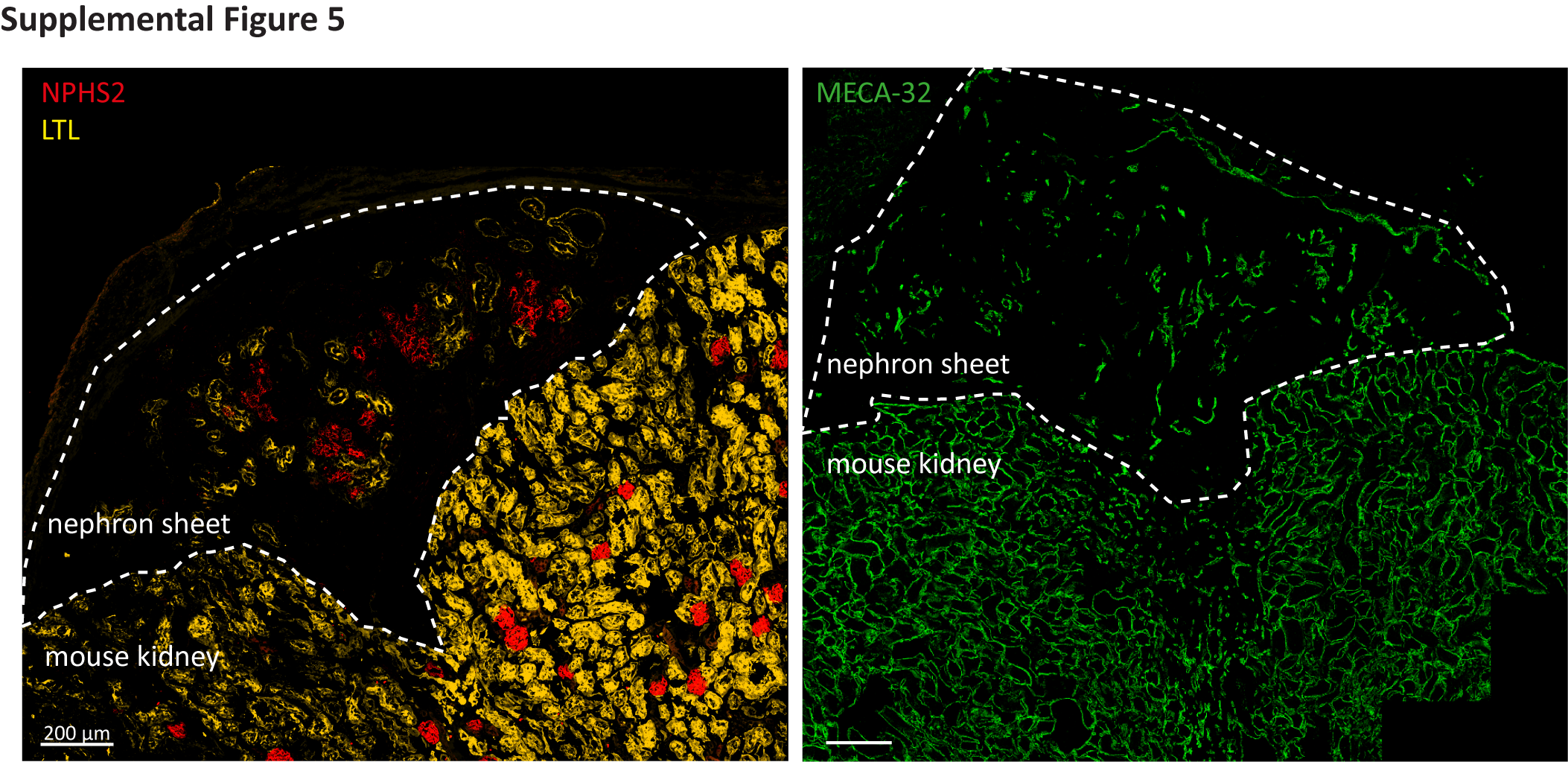

Supplement: Supplementary file 6 — Additional file 6: Fig. S5. Overview of transplanted hiPSC derived nephron sheet. Transplanted biopsy from hiPSC-derived nephron sheet (dotted line, LUMC0072) on the kidney of the recipient mouse. The antibodies for glomerular (NPHS2) and tubular structures (LTL) also recognize these structures in the mouse kidney, but the morphology is different and the hiPSC-derived tissues can be well distinguished from the mouse kidney. MECA-32 only stains mouse endothelial cells. [file 13287_2022_2881_MOESM6_ESM.tif]

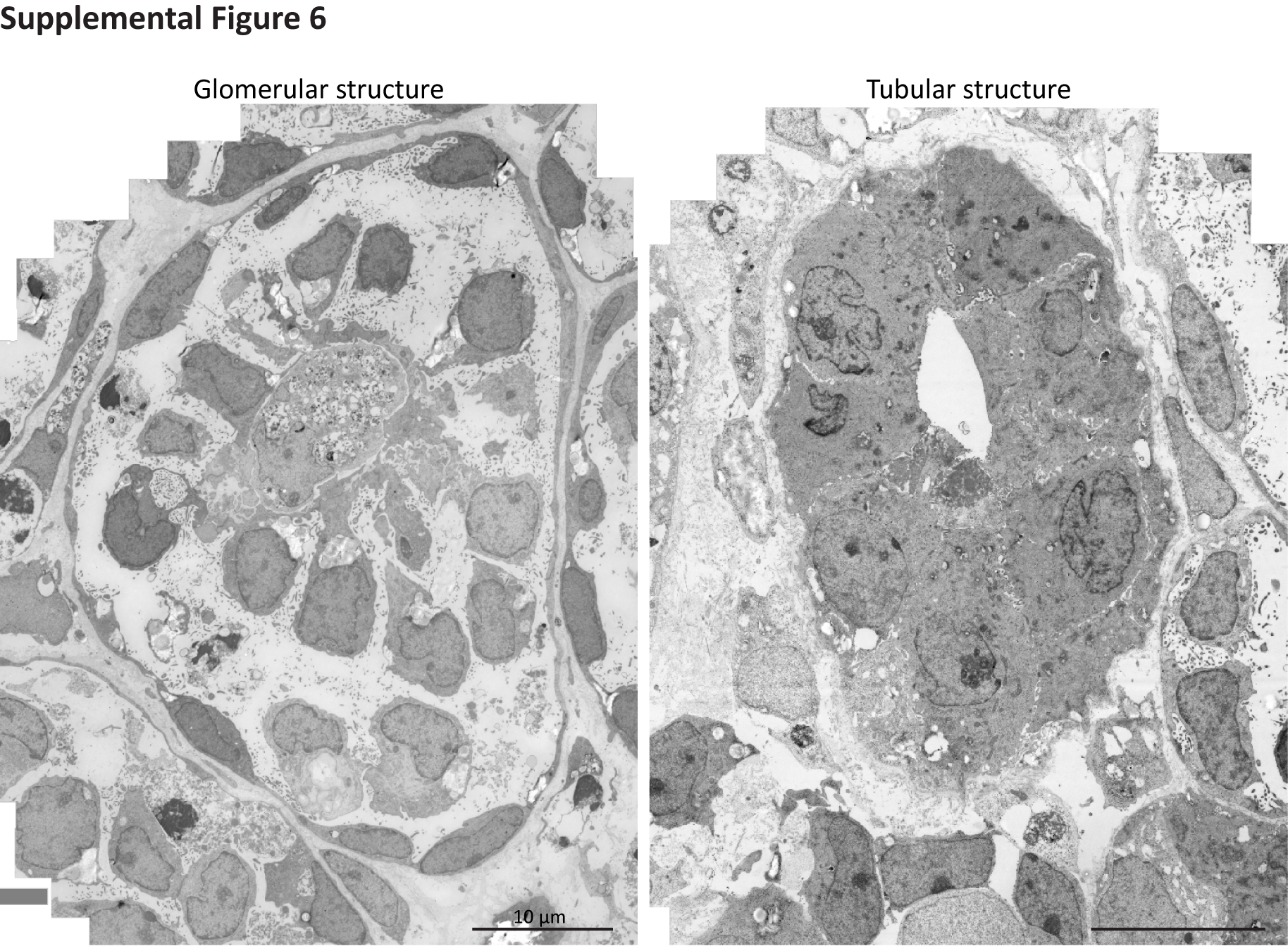

Supplement: Supplementary file 7 — Additional file 7: Fig. S6. hiPSC-derived nephron sheets in vitro at d7 + 31 lack vascularization and are less mature. Transmission electron micrographs of a glomerular and tubular structure in untransplanted nephron sheet. Glomerular structure shows formation of Bowman’s space and centered podocytes anticipating vascularization. The tubular structure is open and epithelial cells are disorganized. [file 13287_2022_2881_MOESM7_ESM.tif]

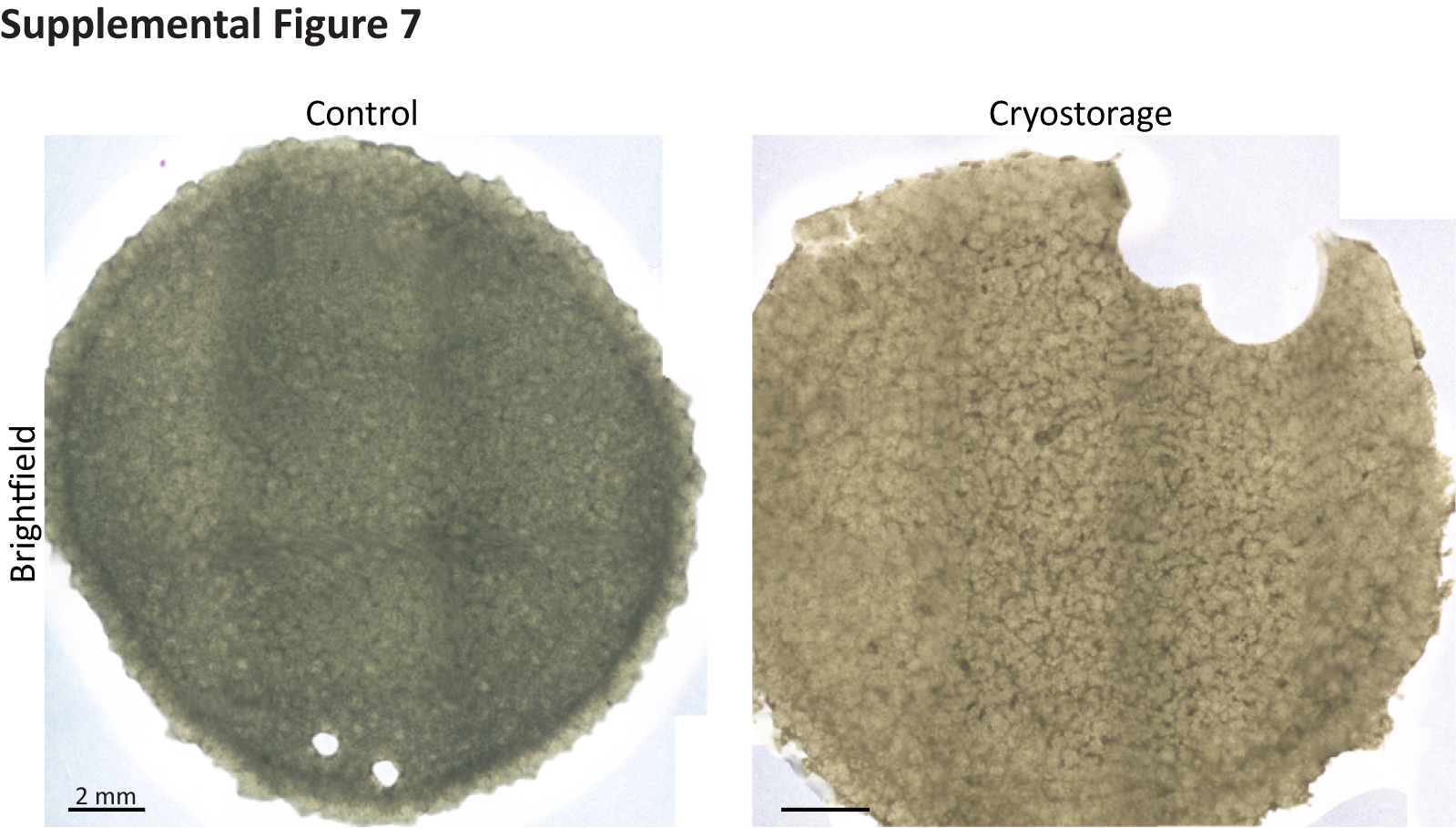

Supplement: Supplementary file 8 — Additional file 8: Fig. S7. Overview of nephron sheets without and with cryopreservation. Brightfield images of hiPSC-derived nephron sheets without (control) and after cryopreservation. [file 13287_2022_2881_MOESM8_ESM.tif]
